# Whole-genome sequence of *Paenibacillus* sp.*,* isolated from soil, Fort Collins, Colorado, USA

**DOI:** 10.1128/MRA.00542-23

**Published:** 2023-09-25

**Authors:** Paris M. Kiehl, Shelby M. Cagle, Traci L. Kinkel, Mark D. Stenglein

**Affiliations:** 1 Department of Microbiology, Immunology, and Pathology, Colorado State University, Fort Collins, Colorado, USA; DOE Joint Genome Institute, Berkeley, California, USA

**Keywords:** bacteriology, genomics, *Paenibacillus*

## Abstract

A whole genome sequence of a *Paenibacillus* sp. bacterium isolated from soil in Fort Collins, Colorado was obtained. This bacterium was of particular interest due to its antibiotic potential against Gram-positive pathogen surrogates.

## ANNOUNCEMENT

This isolate was collected in 2022 as part of the Small World Initiative (1). Soil at Riverbend Natural Area, Colorado, USA (40.5719 N 105.0269 W) was serially diluted in phosphate-buffered saline and plated onto potato dextrose agar (PDA). A colony was streaked to purity on tryptic soy agar (TSA). Colonies were colorless on PDA but pink on TSA. The bacterium produced zones of inhibition against Gram-positive ESKAPE surrogates in a spread patch assay (2).

A TSA colony was used to inoculate a tryptic soy broth (TSB) culture, from which DNA was isolated by phenol-chloroform extraction and ethanol precipitation. 16S ribosomal RNA gene sequence was amplified using Promega PCR Master Mix and 27F/1492R primers (3). The 16S Sanger sequence produced equally scoring BLASTN alignments with 100% identity to >100 *Paenibacillus* species (e.g., MN629116.1). MALDI-TOF mass spectrometry failed to identify the microbe.

For genome sequencing, DNA was isolated from a second TSB culture using Qiagen QiaAmp Fast DNA Tissue Kit. Manufacturer’s protocols were followed unless noted. For Illumina library preparation, DNA was fragmented to an average length of ~400 nt with a Covaris M220 ultrasonicator. DNA size distribution was determined using Agilent 2200 TapeStation. Library molecules were created with Quantabio sparQ DNA kit. Library preparation included end repair and A-tailing, adapter ligation, and library amplification. A BluePippin instrument was used to isolate library molecules of 300–600 bp. Library molarity was determined using Kapa Library Quantification kit (Roche). The library was sequenced on an Illumina MiSeq using a v2 500 cycle sequencing kit to generate 1.6 × 10^6^ 2 × 250 paired-end reads. Nanopore libraries were prepared using the Oxford Nanopore Technologies Rapid Barcoding Kit SQK-RBK004. Libraries were sequenced on a MinION Flongle 9.4.1 flow cell, producing 20,414 reads. Guppy v.6.3.4 was used for high accuracy mode basecalling (4). The nanopore read N50 was 13,371 (5).

Adapters were trimmed with cutadapt v3.5 (6). Default parameters were generally used; full command lines are available at the github repository linked below. Read quality and absence of adapters were assessed using FASTQC v.0.11.9 (7). A hybrid assembly using SPAdes v.3.15.5 (8) produced 26 contigs and 21 scaffolds (9). The largest scaffold was circular by inspection of reads mapped across the ends. Remaining contigs were ≤531 nt long, aligned with high-percent identity to the largest scaffold and were discarded from the assembly. QUAST v.5.2.0 was used to find the contig N50 of 5,045,338 nt (10). Short and long reads were remapped to the genome using bowtie2 v.2.4.5 and minimap2 (11, 12). Samtools v.1.14 was used to determine the coverage depth: 73× for Illumina and 13× for Nanopore (13). The assembly was annotated using Bakta v.1.6.1 (14). The GC content was 40.5%, and 4,536 genes were identified.

## Data Availability

This Whole Genome Shotgun sequence has been deposited in NCBI under accession CP126313.1. The deposited version was reannotated by NCBI using the prokaryotic genome annotation pipeline (15). Reads are available under BioProject accession PRJNA929261 and SRA run IDs SRR23337608 and SRR23337609. Analysis code is available at https://github.com/pkiehl2002/2022_MIP_280A4_final_project.
